# Shining brightly into the future with *Light: Science & Applications*

**DOI:** 10.1038/s41377-025-01938-3

**Published:** 2025-09-18

**Authors:** Xi-Cheng Zhang, Yun-Feng Xiao

**Affiliations:** 1 The Institute of Optics, University of Rochester, Rochester, NY 14627 USA; 2 Frontiers Science Center for Nano-optoelectronics & School of Physics, Peking University, Beijing, 100871 China

**Keywords:** Terahertz optics, Quantum optics

As Co-Editors-in-Chief of *Light: Science & Applications* (*LSA*), we are honored to reflect on the journal’s illustrious history, celebrate its achievements in 2024, and outline our ambitious vision for the future. Launched in March 2012 by the Changchun Institute of Optics, Fine Mechanics and Physics, Chinese Academy of Sciences, and the Chinese Optical Society, in partnership with Nature Portfolio, *LSA* has become a global leader in optics and photonics research. With over 12,000 submissions and 2,200 publications from more than 70 countries and 1500 affiliations, *LSA’*s impact is undeniable, boasting a Journal Impact Factor of 23.4 (JCR 2024), ranking it among the top optics journals worldwide.

In 2024, *LSA* continued its upward trajectory, receiving nearly 2000 submissions and publishing 314 high-impact papers. These contributions spanned cutting-edge topics, including nanophotonics, quantum optics, and advanced sensing technologies, reinforcing our commitment to transformative research. A highlight of the year was the resounding success of the Light Conference 2025, held on June 9-13, 2025. This landmark event attracted over 800 attendees from 26 countries, featuring 7 plenary talks, 22 keynote speeches, 137 invited talks, 2 oral presentations, and 106 posters. The conference showcased groundbreaking research, fostered vibrant discussions, and forged new collaborations, cementing *LSA’s* role as a catalyst for innovation in the global optics community.

Looking forward, *LSA* aims to remain at the forefront of optics and photonics by prioritizing paradigm-shifting research over incremental or technically correct papers. We are committed to maintaining rigorous quality control, as evidenced by our selective publication process. Our goal is to foster innovation through open-access publishing, interdisciplinary collaboration, and rapid dissemination of research. In 2024, *LSA’s* median time to first editorial decision was 6 days, and submission to acceptance was 135 days, demonstrating our unmatched speed and efficiency. Light rejection rate in 2024 is 77%.

We warmly invite researchers to submit their most innovative manuscripts to *LSA*. We seek bold, high-impact work that redefines the field, supported by our robust peer-review process and author-centric initiatives, such as cover art promotion and global outreach. Inspired by the success of the Light Conference 2025, we are committed to nurturing a vibrant platform for scientific exchange. Together, let us illuminate the future of light science with *Light: Science & Applications*.

